# An ABA down-regulated bHLH transcription repressor gene, *bHLH129* regulates root elongation and ABA response when overexpressed in Arabidopsis

**DOI:** 10.1038/srep17587

**Published:** 2015-12-02

**Authors:** Hainan Tian, Hongyan Guo, Xuemei Dai, Yuxin Cheng, Kaijie Zheng, Xiaoping Wang, Shucai Wang

**Affiliations:** 1 Key Laboratory of Molecular Epigenetics of MOE, Northeast Normal University, Changchun, Jilin 130024, China

## Abstract

Plant hormone abscisic acid (ABA) plays a crucial role in modulating plant responses to environmental stresses. Basic helix-loop-helix (bHLH) transcription factors are one of the largest transcription factor families that regulate multiple aspects of plant growth and development, as well as of plant metabolism in Arabidopsis. Several bHLH transcription factors have been shown to be involved in the regulation of ABA signaling. We report here the characterization of bHLH129, a bHLH transcription factor in Arabidopsis. We found that the expression level of *bHLH129* was reduced in response to exogenously applied ABA, and elevated in the ABA biosynthesis mutant *aba1-5*. Florescence observation of transgenic plants expressing *bHLH129-GFP* showed that bHLH129 was localized in the nucleus, and transient expression of *bHLH129* in protoplasts inhibited reporter gene expression. When expressed in Arabidopsis under the control of the *35S* promoter, *bHLH129* promoted root elongation, and the transgenic plants were less sensitivity to ABA in root elongation assays. Quantitative RT-PCR results showed that ABA response of several genes involved in ABA signaling, including *ABI1*, *SnRK2.2*, *SnRK2.3* and *SnRK2.6* were altered in the transgenic plants overexpressing *bHLH129.* Taken together, our study suggests that bHLH129 is a transcription repressor that negatively regulates ABA response in Arabidopsis.

Basic helix-loop-helix (bHLH) transcription factors are one of the largest transcription factor families find in almost all organisms, including fungi, animals and plants^1^. In Arabidopsis, bHLH transcription factors regulate multiple aspects of plant growth and development, as well as plant metabolism^2^. For example, the bHLH transcription factors GLABRA3 (GL3), ENHANCER OF GLABRA3 (EGL3) and TRANSPARENT TESTA 8 (TT8) are involved in the regulation of trichome and root hair formation, hypocotyl stomata patterning, mucilage and anthocyanin biosynthesis in Arabidopsis^3^,^4^,^5^,^6^,^7^, by interacting with the WD40-repeat protein TRANSPARENT TESTA GLABRA1 (TTG1) and several different R2R3 MYB proteins to form multiple MYB-bHLH-WD40 (MBW) transcription activator complexes^8^,^9^, FLOWERING BHLH (FBH) transcription activators FBH1, FBH2, FBH3 and FBH4 control flowering time by regulating the expression of the photoperiodic flowering regulator gene *CONSTANS* (*CO*)^10^, and PHYTOCHROME INTERACTING FACTOR 3-LIKE 5 (PIL5) regulates seed germination by activating the expression of a C3H-type zinc finger gene *SOMUNS* (*SOM*)^11^,^12^.

In addition to regulate metabolism and plant growth and development, bHLH transcription factors are also involved in the regulation of different signaling pathways including light signaling and plant hormone signaling. For example, REDUCED SENSITIVITY TO FAR-RED LIGHT 1 (REP1), PHYTOCHROME-INTERACTING BHLH FACTORS (PIFs) and bHLH135 are involved in the regulation of light signaling^13^,^14^,^15^,^16^, MYC2 is a master regulator of jasmonate (JA) signaling and it regulates crosstalk between JA and other plant hormones^17^, and typical bHLH proteins including ACTIVATION-TAGGED BRI1(BRASSINOSTEROID-INSENSITIVE 1)-SUPPRESSOR 1 (ATBS1) and PACLOBUTRAZOL RESIS-TANCE1 (PRE1) are involved in the regulation of brassinosteroid signaling^18^,^19^.

Abscisic acid (ABA) is one of the earliest identified plant hormones^20^. Though it regulates some processes of plant growth and development such as seed germination, seed maturation and bud dormancy, ABA is largely recognized by its roles in the regulation of plant responses to environmental stresses including drought, cold, heat and salinity^21^,^22^,^23^. In these processes, ABA functions through a complex web of signaling networks, in which many components including receptors, phosphatases, protein kinases, E3 ligases and transcription factors are involved^21^,^22^,^23^,^24^.

Several bHLH transcription factors have been shown to be involved in ABA signaling, and the ways how bHLH transcription factors are involved in ABA signaling are different. INDUCER OF CBF EXPRESSION 2 (ICE2) induces ABA biosynthesis^25^, and overexpression of *bHLH122* increased cellular ABA levels^26^. The expression of both *ABA-INDUCIBLE BHLH-TYPE TRANSCRIPTION FACTOR* (*AIB*) and *ANDROGEN-INDUCIBLE GENE 1* (*AtAIG1*) are induced by ABA^27^,^28^, however, AIB positively regulates ABA response in Arabidopsis^28^, whereas AtAIG1 can bind to the E-box sequence present in the promoter regions of many ABA-responsive genes, and negatively regulates ABA response^27^. AtMYC2 interacts with R2R3 MYB transcription factor AtMYB2 to activate the expression of ABA response gene *RESPONSIVE TO DEHYDRATION 22* (*RD22*)^29^, while AtbHLH112 functions as a transcription activator and binds to GCG-box or E-box motifs to induce proline biosynthesis and ROS scavenging pathway^30^. On the other hand, ABA can induce the phosphorylation of three bHLH transcription factors, including ABA-RESPONSIVE KINASE SUBSTRATE 1 (AKS1), AKS2 and AKS3, leading to the repression of their transcriptional activities^31^.

Here we report the characterization of bHLH129, an Arabidopsis bHLH transcription factor. We showed that expression of *bHLH129* was down-regulated by exogenously applied ABA. Protoplast transient transfection assay results indicated that bHLH129 is a transcription repressor. When expressed in Arabidopsis, bHLH129 promoted root elongation, and the transgenic plants overexpressing *bHLH129* were less sensitive to ABA. In addition, we showed that ABA response of some ABA signaling genes including *ABA INSENSITIVE 1* (*ABI1*), *SNF1-RELATED PROTEIN KINASE 2.2* (*SnRK2.2*), *SnRK2.3* and *SnRK2.6* were altered in the transgenic plants.

## Results

### Expression of bHLH129 is down-regulated by ABA

Arabidopsis bHLH transcription factors have been shown to regulate plant metabolism, as well as multiple aspects of plant growth and development^2^. Several bHLH transcription factors are reported to regulate ABA response^25^,^28^,^29^,^30^. Our previously microarray data showed that expression of *bHLH129* (*At2g43140*) is regulated by ABA^32^, suggesting that bHLH129 may involve in the regulation of ABA response.

**Figure 1:**
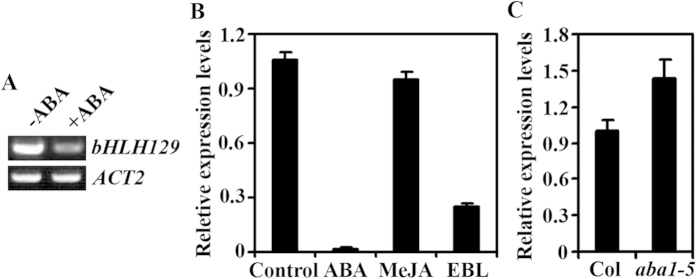
Expression of *bHLH129* is negatively regulated by ABA. (**A**) Expression of *bHLH129* in Arabidopsis seedlings in response to ABA treatment. RNA was isolated from ABA treated Arabidopsis seedlings and RT-PCR was used to examine the expression of *bHLH129*. Expression of *ACT2* was used as a control. (**B**) Quantitative RT-PCR (qRT-PCR) analysis of *bHLH129* expression in response to ABA, methyl jasmonate (MeJA), and 2,4-epibrassinolide (EBL) treatments. RNA was isolated from ABA, methyl jasmonate, or 2,4-epibrassinolide treated Arabidopsis seedlings, and qRT-PCR was used to examine the expression of *bHLH129*. Expression of *ACTIN2* was used as a reference gene, and expression of *bHLH129* in the absence of ABA was set as 1. Data represent the mean ± standard deviation (SD) of three replicates. (**C**) Expression of *bHLH129* in *aba1-5* mutants. RNA was isolated from 12-day-old Col and *aba1-5* mutant seedlings and qRT-PCR was used to examine the expression of *bHLH129*. Expression of *ACT2* was used as a reference gene, and expression of *bHLH129* in Col wild type was set as 1. Data represent the mean ± standard deviation (SD) of three replicates.

To investigate the possible roles of bHLH129 in ABA response in Arabidopsis, we examined if the expression of *bHLH129* is regulated by ABA. We first examined if ABA treatment will affect the expression of *bHLH129*. Arabidopsis seedlings were treated with ABA for 3 h, RNA was isolated and RT-PCR or quantitative RT-PCR (qRT-PCR) was used to examine the expression of *bHLH129*. As shown in Fig. 1A, expression level of *bHLH129* in Arabidopsis seedlings was dramatically reduced in response to exogenously applied ABA. Quantitative RT-PCR results showed that transcript levels of *bHLH129* were decreased about 60-fold and 4-fold, respectively after ABA and 2,4-epibrassinolide treatments, but remained largely unchanged after methyl jasmonate treatment (Fig. 1B). We then examined if expression of *bHLH129* is altered in *aba1-5* (Fig. 1C), an ABA biosynthesis mutant^33^. We found that the transcript levels of *bHLH129* increased ~1.4-fold in the *aba1-5* mutant seedlings. These results suggest that *bHLH129* is an ABA response gene and its expression is down-regulated by ABA.

### Expression patterns of *bHLH129* in Arabidopsis

**Figure 2:**
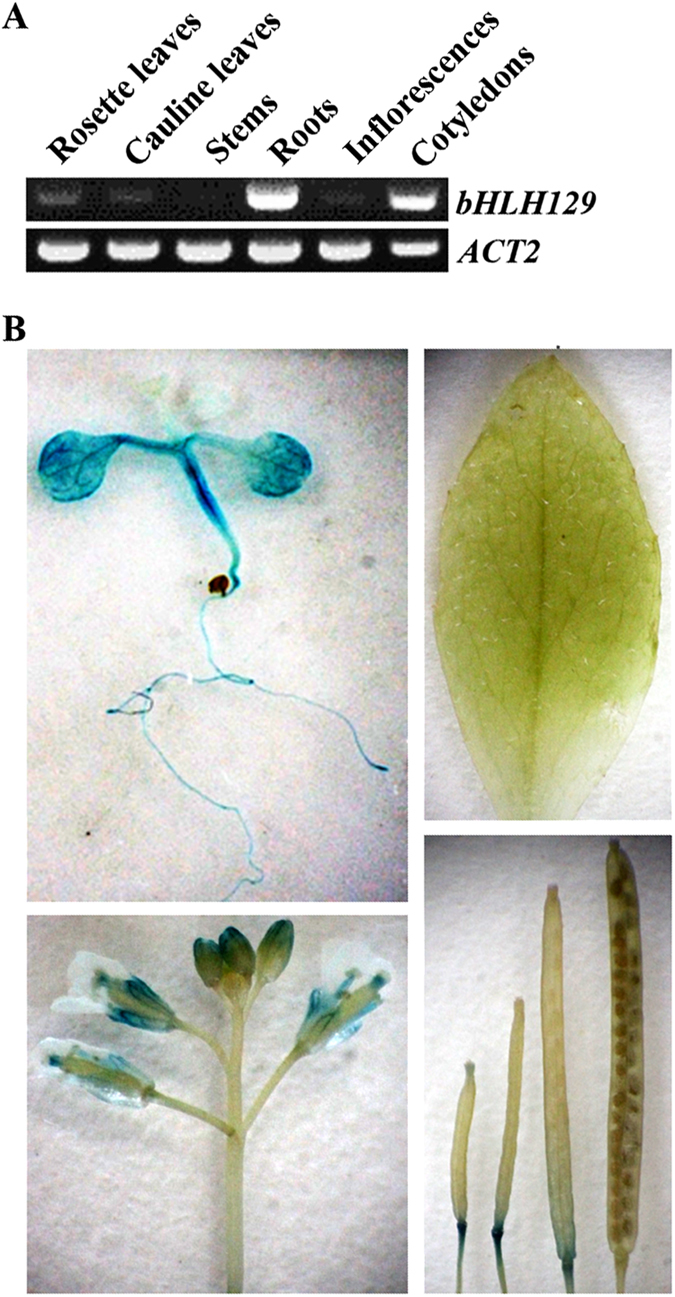
Expression pattern of *bHLH129*. (**A**) Expression of *bHLH129* in different tissues and organs of the Col wild type Arabidopsis. RNA was isolated from different tissues and organs of the Col wild type Arabidopsis and RT-PCR was used to examine the expression of *bHLH129*. Expression of *ACT2* was used as a control. (**B**) Expression of *bHLH129p:GUS* in 14-day-old seedlings (upper panel, left), rosette leaves (upper panel, right), infloresences (lower panel, left) and siliques at different developmental stages (lower panel, right). X-Gluc (5-bromo-4-chloro-3-indolyl β-D-glucuronide) was used for histochemical staining of GUS activity.

To examine the expression patterns of *bHLH129*, different tissues and organs were collected from Arabidopsis seedlings and mature plants, and RT-PCR was used to examine the expression of *bHLH129* in the samples collected. We found that transcripts of *bHLH129* were detectable in all tissues and organs examined but stems, with relative higher expression levels detected in roots and cotyledons (Fig. 2A).

To examine the expression patterns of *bHLH129* in more details, we generated reporter transgenic plants by transforming wild-type plants with *bHLH129p:GUS* construct, and examined *GUS* expression in the transgenic plants. We found that, consistent with the RT-PCR results, *bHLH129p:GUS* was highly expressed in the roots and cotyledons, but not stems (Fig. 2B). Higher expression level of *bHLH129p:GUS* was also observed in hypocotyls and some flower organs (Fig. 2B). We also found that *bHLH129p:GUS* was expressed in young rosette leaves and the lower part of young siliques, but not mature rosette leaves and old siliques (Fig. 2B), suggesting that expression of *bHLH129p:GUS* appears to be developmentally regulated.

### bHLH129 is a transcription repressor

**Figure 3:**
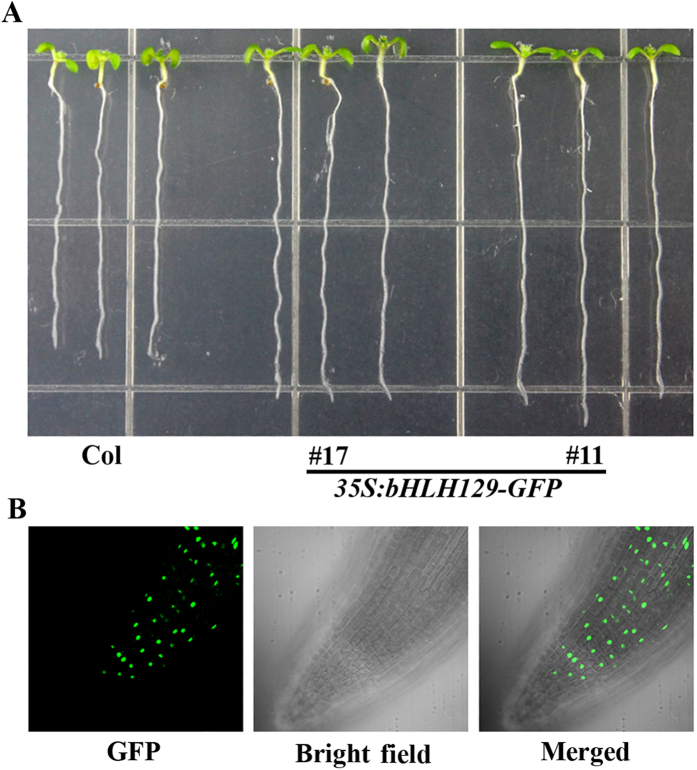
Subcellular localization of bHLH129. (**A**) Seven-day-old Col wild type and *35S:bHLH129-GFP* transgenic plant seedlings. (**B**) Subcellular localization of bHLH129. Root tips of the *35S:bHLH129-GFP* transgenic plant seedlings were examined under a florescence microscope. Left panel: GFP channel, middle panel: bright field image, right panel: merged.

Some bHLH transcription factors such as GL3, AtbHLH112 and AtMYC2 have been shown to be transcription activators^30^,^34^, whereas some others such as JASMONATE-ASSOCIATED MYC2-LIKE1 (JAM1), JAM2 and JAM3 are repressors^35^,^36^. To explore bHLH129’s functions in Arabidopsis, we decided to examine if bHLH129 functions as transcription activator or repressor. We first examined bHLH129’s subcellular localization by generating *bHLH129-GFP* transgenic plants and examining GFP florescence in the transgenic plants. The *bHLH129-GFP* transgenic plant seedlings have longer primary root when compared with that in Col wild type seedlings (Fig. 3A), a phenotype similar to that of the *bHLH129* transgenic plants (see next section for details), suggesting that the bHLH129-GFP was functional. By examining GFP florescence in the root of the *bHLH129-GFP* transgenic plant seedlings obtained, we found that bHLH129 is predominantly localized in the nucleus (Fig. 3B).

**Figure 4:**
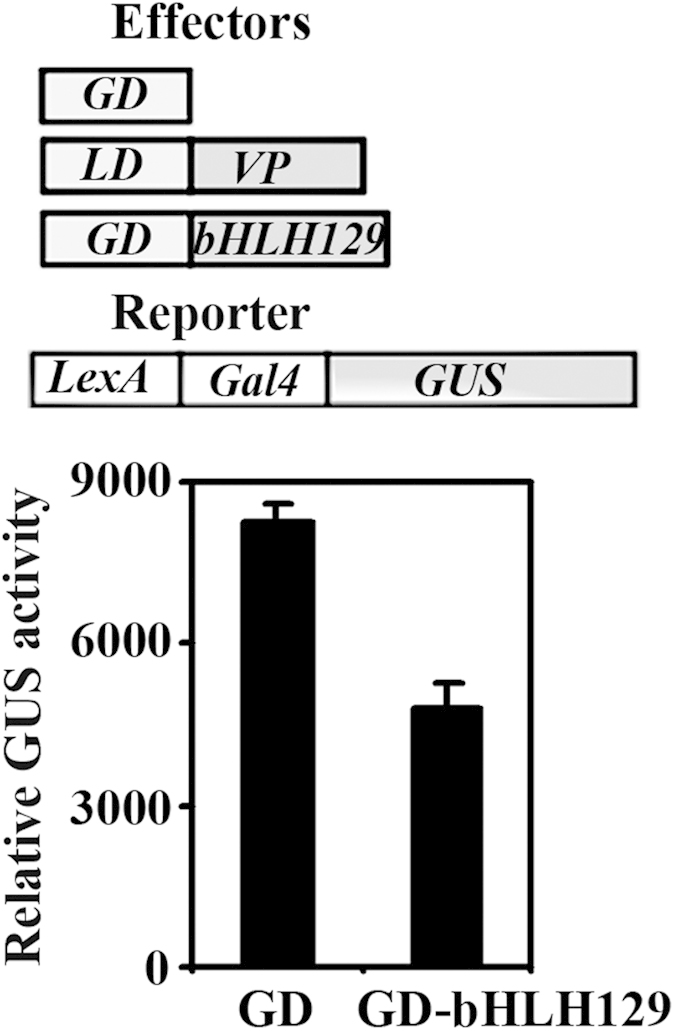
bHLH129 is a transcription repressor. Effectors and reporter (diagrammed on the top of the figure) were co-transfected into protoplasts isolated from Col wild type, and the transfected protoplasts were incubated in darkness for 20-22 h before GUS activity was assayed. Data represent the mean ± SD of three replicates.

We then examined transcriptional activities of bHLH129 using protoplast transient transfection assays. Plasmids of activator gene *LD-VP*, effector gene *GD-bHLH129* or control gene *GD*, and the reporter gene *LexA-Gal4:GUS* were co-transfected into protoplasts, and GUS activities were measured after incubation of the transfected protoplasts overnight at darkness. In this system, the LD-VP activator can be recruited to the *LexA* DNA binding site thus activating the reporter gene, whereas GD control or GD-bHLH129 can be recruited to the *Gal4* DNA binding site of the reporter gene. If bHLH129 functions as a transcription repressor, co-transfection of *GD-bHLH129* will result in repression of the reporter gene activated by LD-VP. As shown in Fig. 4, co-transfection of the activator gene *LD-VP* and the control gene *GD* activated the reporter gene, while co-transfection of the effector gene *GD-bHLH129* resulted in repression of the reporter gene, indicating that bHLH129 is a transcription repressor.

### Overexpression of *bHLH129* in Arabidopsis promotes root elongation

Having shown that expression of *bHLH129* is down-regulated by ABA (Fig. 1), and bHLH129 functions as a transcription repressor (Fig. 4), we further explored the functions of bHLH129 by generating transgenic plants expressing *bHLH129* under the control of the *35S* promoter (*35S:bHLH129*), isolating *bHLH129* knock-out mutants, and examining phenotypes of the transgenic plants and the mutants obtained.

**Figure 5:**
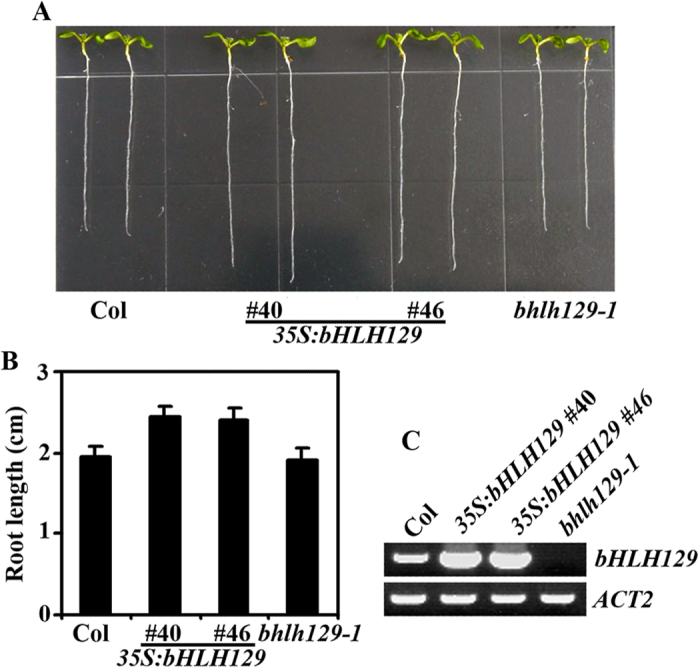
Phenotypes of Arabidopsis transgenic plant seedlings expressing *bHLH129*. (**A**) Seven-day-old Col wild type and transgenic plant seedlings expressing *bHLH129* under the control of the *35S* promoter. (**B**) Primary root length of 7-day-old wild type and transgenic plants. Data represent the mean ± SD of 19-23 seedlings. (**C**) Expression of *bHLH129* in the transgenic plants. RNA was isolated from Col wild type, *bHLH129* transgenic, and *bhlh129-1* mutant seedlings and RT-PCR was used to examine the expression of *bHLH129*. Expression of *ACT2* was used as a control.

Plant overexpressing *bHLH129* was obtained by transforming Col wild type plants with the *35S:bHLH129* construct, and *bHLH129* knock-out mutant *bhlh129-1* was isolated from a SALK T-DNA insertion line (SALK_041780) obtained from ABRC. As shown in Fig. 5A, *bHLH129* transgenic Arabidopsis seedlings have increased primary root length when compared with that of the wild type seedlings, whereas the primary root length of the *bhlh129-1* mutant seedlings were largely indistinguishable from that of the wild type seedlings. Quantitative analysis showed that there was an ~20% increase in the primary root length of the transgenic plants (Fig. 5B). Overexpression of *bHLH129* in the transgenic plants and knock-out of *bHLH129* in the *bhlh129-1* mutant were confirmed by RT-PCR (Fig. 5C).

Transgenic plant seedlings expressing GFP tagged bHLH129 (*35S:bHLH129-GFP*) also resulted in increased primary root length (Fig. 3A), indicating that bHLH129-GFP is functional, thus the plants were used to examine subcellular localization of bHLH129 (Fig. 3B).

### Transgenic plants overexpressing *bHLH129* are less sensitive to ABA

**Figure 6:**
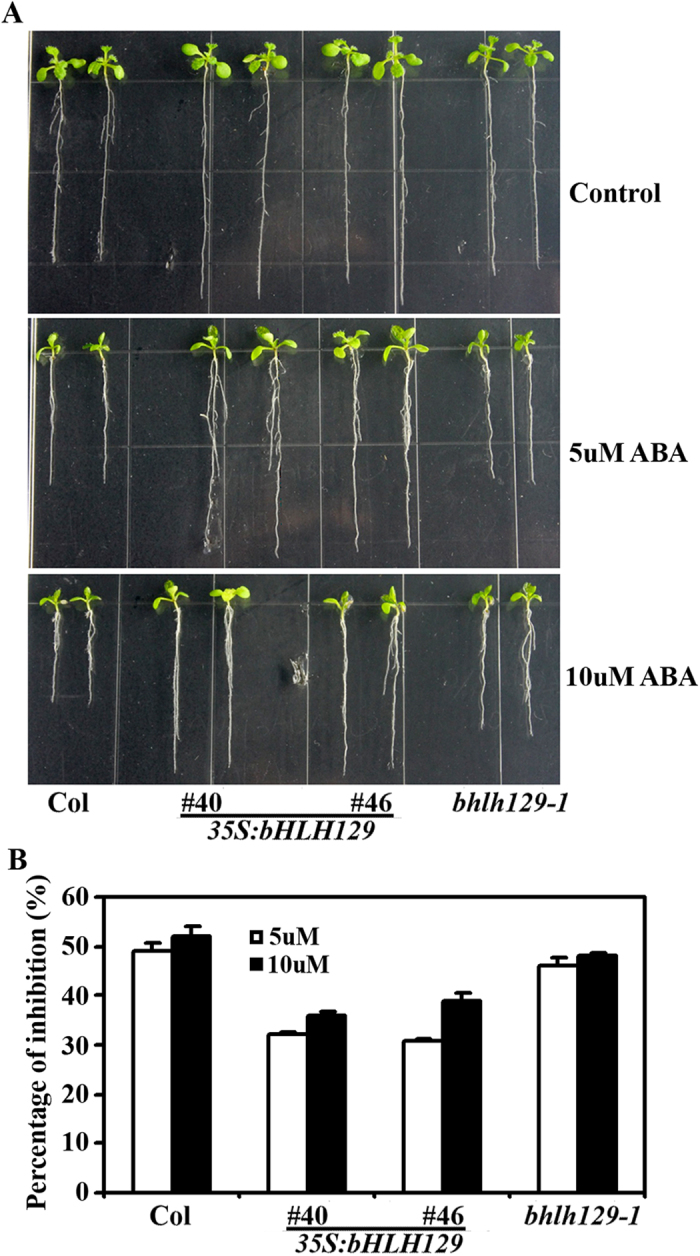
Effects of ABA on root elongation in Col wild type and *bHLH129* transgenic plant seedlings. (**A**) Fourteen-day-old seedlings on vertical plates. Seedlings were grown vertically on 1/2 MS plates for 4 d, and then transferred to plates containing 5 μM or 10 μM ABA and grown for 10 d. (**B**) Root elongation inhibition by ABA. Length of new elongated roots was measured, and percentage of inhibition was calculated. Data represent means ± SD of 9-11 seedlings.

Because *bHLH129* is an ABA response gene (Fig. 1), we further examined if bHLH129 is involved in regulating ABA response by examining ABA responsiveness of the *bHLH129* transgenic plants obtained. ABA inhibited root elongation was used to analyze ABA responsiveness in the transgenic plants. Four-day-old seedlings of wild-type and the *bHLH129* transgenic plants grown on vertical 1/2 MS plates were transferred to new 1/2 MS plates at the presence and absence of ABA and grown vertically for additional 10 days. The primary root length was measured and percentage of root inhibition was calculated. As shown in Fig. 6, Arabidopsis seedlings over-expressing *bHLH129* was less sensitive to ABA treatments. The root elongation of wild-type seedlings was inhibited about 50% by 5 μM ABA, whereas that of *bHLH129* transgenic plants was only about 30% (Fig. 6B). Root elongation was further inhibited by 10 μM ABA, however, the *bHLH129* transgenic plant seedlings were still less sensitive to ABA treatments when compared with that of the wild type seedlings (Fig. 6). On the other hand, a near wild type response to ABA treatments was observed with the *bhlh129-1* mutant seedlings (Fig. 6).

### ABA response of some ABA signaling pathway genes is altered in the transgenic plants overexpressing *bHLH129*

**Figure 7:**
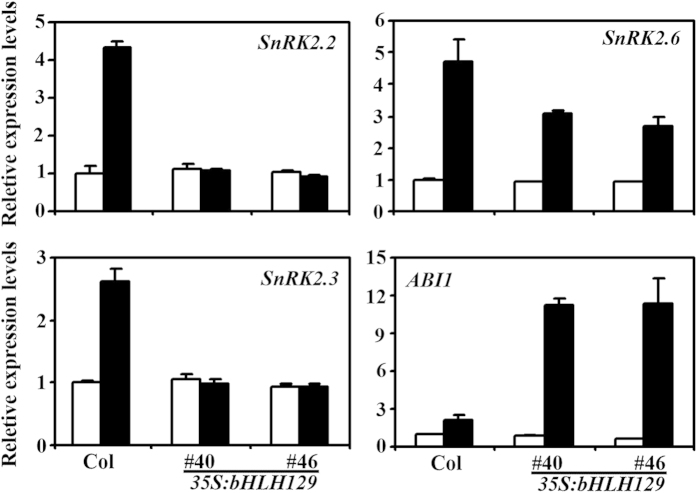
Expression of the genes involved in ABA signaling in wild type and *bHLH129* transgenic plant seedlings. RNA was isolated from ABA treated Col wild type and *bHLH129* transgenic plant seedlings, and qRT-PCR was used to examine the expression of the genes involved in ABA signaling. Expression of *ACT2* was used as a reference gene, and expression of corresponding genes in Col wild type seedlings in the absence of ABA was set as 1. Data represent the mean ± SD of three replicates. White bar, absence of ABA; black bar, presence of ABA.

ABA signaling is regulated by several different types of proteins, including the central regulators PROTEIN PHOSPHATASE 2C (PP2C) A-group proteins and SUCROSE NONFERMENTING 1 (SNF1)-RELATED PROTEIN KINASES (SnRK2s)^21^,^22^,^23^,^24^. To investigate why the *bHLH129* transgenic plants were less sensitive to ABA treatment when compared to the Col wild type, we examined the expression of some ABA signaling regulator genes including *PP2C* genes and *SnRK2* genes in the *bHLH129* transgenic plants. As shown in Fig. 7, the expression levels of *SnRK2* gene *SnRK2.2*, *SnRK2.3* and *SnRK2.6*, and *PP2C* gene *ABA INSENSITIVE 1* (*ABI1*) in the transgenic plant seedlings were largely unaffected. When compared to that in the Col wild type plant seedlings, however, their response to ABA was altered in the transgenic plants overexpressing *bHLH129* (Fig. 7). An ~2–5 folds induction of the above mentioned genes in response to ABA was observed in the Col wild type seedlings (Fig. 7). In the *bHLH129* transgenic seedlings, the expression of *SnRK2.2* and *SnRK2.3* was no longer induced by ABA treatment. On the other hand, expression levels of *SnRK2.6* in response to ABA in the transgenic plant seedlings was reduced to about two-third of that in the wild type seedlings, whereas the expression levels of *ABI1* was increased about 4 folds (Fig. 7).

## Discussion

There are 162 genes in Arabidopsis encoding bHLH transcription factors^37^. So far only a few of them have been reported to be involved in the regulation of ABA response^25^,^28^,^29^,^30^. Our results here show that *bHLH129* is an ABA response gene, it encodes a transcription repressor, and is involved in the regulation of ABA response in Arabidopsis.

Among the several bHLH transcription factor genes that involve in the regulation of ABA response in Arabidopsis, *AIB* and *AtAIG1* have been shown to be up-regulated by ABA^27^,^28^, overexpression of *bHLH122* increases cellular ABA levels, but *bHLH122* itself is not induced by ABA^26^. Two different pieces of evidence suggest that *bHLH129* is an ABA response gene, but its expression is down-, rather than up-regulated by ABA: expression levels of *bHLH129* were reduced in the presence of exogenously applied ABA, and elevated in the ABA biosynthesis mutant *aba1-5* (Fig. 1).

Several bHLH transcription factors that regulate ABA response in Arabidopsis including bHLH112 and AtMYC2 have been shown to be transcription activator^29^,^30^. AIB/JIM1 is initially showed as a transcription activator in yeast cells^28^, but assays in plant cells suggest that AIB/JIM1 and its homologues JAM2 and JAM3 function as transcription repressor, and they are involved in the regulation of JA signaling^35^,^36^. We found that bHLH129 is a nucleus protein (Fig. 3), when transient expressed in protoplasts, it repressed the expression of the reporter gene activated by a transcription activator, indicating that bHLH129 is a transcription repressor.

When overexpressed in Col wild type plants, *bHLH129* promoted root elongation (Fig. 5), and the *bHLH129* transgenic plants were less sensitive to ABA in root elongation assays (Fig. 6), suggesting that bHLH129 is a negative regulator of ABA response. Considering that ABA down-regulate the expression of *bHLH129*, while bHLH129 negatively regulates ABA response, it is likely that bHLH129 plays a role under normal growth conditions when ABA response should not be triggered. In Col wild type plants, application of ABA resulted in decreased expression of *bHLH129*, the plants showed a normal ABA response. In the *35S:bHLH129* transgenic plants, expression of *bHLH129* is no longer down-regulated by ABA, thus the plants showed a reduced response to ABA treatment. However, *bHLH129* knock-out mutant *bhlh129-1* is morphological similar to Col wild type plants (Fig. 5), and it showed a near wild type response to ABA. This may due to redundancy functions of other related bHLH transcription factors.

The mechanisms bHLH transcription factors used to regulate ABA signaling are different, some of them including ICE2 and bHLH122 affect ABA biosynthesis^25^,^26^, and some others such as AtAIG1 and AtMYC2 directly regulate the expression of ABA response genes^27^,^29^.

PP2Cs and SnRK2s are key central regulators in ABA signaling pathway^21^,^22^,^23^,^24^. We found that although the expression levels of *PP2Cs* and *SnRK2s* genes including *ABI1*, *SnRK2.2*, *SnRK2.3* and *SnRK2.6* in the transgenic plants overexpressing *bHLH129* were largely unaffected when compared with that in the Col wild type plants at the absence of ABA, however, their response to ABA was altered (Fig. 7), indicating that bHLH129 may regulate ABA response through regulating the expression of some ABA signaling pathway genes. Because bHLH129 function as a transcription repressor (Fig. 4), and ABA response of *SnRK2s* genes in the transgenic plants was repressed, while that of *PP2C* gene *ABI1* was enhanced (Fig. 7), it is very unlikely that all these genes were sonly regulated by bHLH129. Considering that bHLH transcription factors have been reported to interact with other transcription factors to regulate several different processes including cell fate determination, mucilage and anthocyanin biosynthesis, and to regulate the expression of ABA response gene *RD22*^8^,^9^,^29^, bHLH129 may interact with other transcription factors to regulate ABA response in Arabidopsis. In any case, it will be of great interest to find out how bHLH129 regulates the expression of those genes, and why the effects of bHLH129 on the expression of ABA signaling pathway genes can only be seen in the presence of ABA.

Never the less, our results showed that expression of *bHLH129* is down-regulated by ABA, bHLH129 is a transcription repressor, and it regulated root elongation and ABA response when overexpressed in Arabidopsis, possibly by regulating the expression of some ABA signaling pathway genes.

## Methods

### Plant materials and growth conditions

Arabidopsis (*Arabidopsis thaliana*) ecotype Columbia (Col-0) was used for plant transformation and protoplasts isolation. The *bhlh129-1* mutant was isolated from a SALK T-DNA insertion line (SALK_041780) obtained from ABRC.

To obtain seedlings for phenotypic analysis and ABA treatment, Arabidopsis seeds were sterilized and sown on ½ MS (Murashige & Skoog) plates with vitamins (PlantMedia) and 1% (w/v) sucrose, solidified with 0.6% (w/v) phytoagar (PlantMedia). The plates were kept at 4 °C in darkness for 2 days before moved into a growth room. To obtain plants for plant transformation and protoplasts isolation, Arabidopsis seeds were sown directly into soil pots and kept in a growth room. The growth condition in the growth room was set with a temperature at 22 °C and a photoperiod with 16 h/8 h (light/dark) at a light density of approximately 120 μmol m^−2^s^−1^.

### ABA treatment and root elongation assay

To examine the expression of *bHLH129* and genes involved in ABA signaling in response to ABA, 12-day-old Col wild type seedlings were treated with 10 μM ABA, 200 nM methyl jasmonate, or 100 nM 2,4-epibrassinolide for 3 h in darkness on a shaker at 40 rpm. Samples were frozen in liquid N_2_ and kept at −80 °C for RNA isolation.

To examine ABA sensitivity of the *bHLH129* transgenic plant seedlings, 4-day-old Col wild type and *bHLH129* transgenic plant seedlings grown on vertical plates were transferred into new plates containing either 5 μM or 10 μM ABA and grown vertically. Pictures were taken 10 days after transfer, root length was measured using Image J software, and percentage of inhibition was calculated. The experiments were repeated three times, and data from a representative experiment was presented.

### DNA and RNA isolation, RT-PCR and quantitative RT-PCR (qRT-PCR)

DNA and total RNA from Arabidopsis seedlings were isolated as described previously^9^,^38^,^39^,^40^. cDNA was synthesized using 2 μg total RNA by Oligo(dT)-primed reverse transcription using the EazyScript First-Strand DNA Synthesis Super Mix (TransGen Biotech).

RT-PCR was used to examine the expression of *bHLH129*, qRT-PCR was used to examine the expression of *bHLH129* and genes involved in ABA signaling pathway. Arabidopsis gene *ACTIN2* (*ACT2*) was used as a control for RT-PCR and qRT-PCR. Primers used for RT-PCR and qRT-PCR analysis of *bHLH129* and genes involved in ABA signaling pathway are: *bHLH129*, 5′-TTTCTCTAGGACGGCCAAAC-3′ and 5′-GATGGCTACTACTCCCACTAGA-3′, *SnRK2.2*, 5′-CCGATTATGCACGACAGTGA-3′ and 5′-CAAGCTCCTTGGTGACTCTATC-3′, *SnRK2.3*, 5′-GAAGATCCAGAAGAGCCAAGAG-3′ and 5′-CCGTATGTCATCAGGGATTGAG-3′, *SnRK2.6*, 5′-CGGGCCAAAGCATAGAAGAA-3′ and 5′-CCAAGCTTCCTGTGAGGTAATG-3′. The primers for qRT-PCR examination of *ABI1*, and for RT-PCR and qRT-PCR examination of *ACT2* have been described previously^9^,^40^,^41^.

### Constructs

The reporter construct *LexA-Gal4:GUS*, and effector constructs *GD* and *LD-VP* used for protoplast transfection have been described previously^42^,^43^.

To generate GD tagged bHLH129 construct for protoplast transfection assays and HA tagged bHLH129 construct for plant transformation, the full-length open-reading frame (ORF) of *bHLH129* was amplified by RT-PCR using RNA isolated from the Arabidopsis seedlings, and cloned in frame with an N-terminal GD or HA tag into the *pUC19* vector under the control of the *35S* promoter^44^,^45^. HA tagged bHLH129 construct in *pUC19* was then digested with proper enzymes, and subcloned into the binary vector *pPZP211*^46^.

To generate GFP tagged construct for subcellular localization analysis of bHLH129, the ORF of *bHLH129* was amplified and cloned in frame with a C-terminal GFP tag into the *pUC19*, the construct obtained was then digested with proper enzymes, and subcloned into the binary vector *pPZP211* for plant transformation.

To generate *bHLH129p:GUS* (*β-glucuronidase*) construct for plant transformation, a 2514 bp DNA fragment immediately before the start condon of *bHLH129* was amplified by PCR using DNA isolated from Arabidopsis seedlings, and used to replace the *OFP1* promoter in the *pPZP211OFP1p:GUS* construct^43^.

The primers used to generate *GD/HA-bHLH129* construct are 5′-CAACATATGTACCCTCCTAATTCCTC-3′ and 5′-CAAGAGCTCTCATCGCTTCTTGCATGC-3′, the primers used to generate *bHLH129-GFP* construct are 5′-CAACATATGTACCCTCCTAATTCCTC-3′ and 5′-CAAGAGCTCTCGCTTCTTGCATGC-3′, and the primers used for make *bHLH129p:GUS* construct are 5′-CAACTGCAGCATGATCGGTCTGATTC-3′ and 5′-CAAGAGCTCGAAACCGGAAAAGAAAAACCC-3′.

### Plant transformation and transgenic plants selection

About 5-week-old plants with several mature flowers on the main inflorescence were used for transformation by using the floral dip method^47^. Transgenic plants were selected by grown T1 seeds on 1/2 MS plates containing 50 μg/ml kanamycin and 100 μg/ml carbenicillin. For each construct, at least 5 transgenic lines with similar phenotypes were obtained, and represent homozygous T3 or T4 plants were used for further analysis.

### Plasmid DNA isolation, protoplasts isolation, transfection and GUS activity assays

Plasmids preparation, protoplasts isolation, transfection and GUS activity assay were performed as described previously^9^,^39^,^40^,^43^,^44^,^45^,^48^. In brief, reporter and effector plasmid DNA were isolated using the GoldHi EndoFree Plasmid Maxi Kit (CWbiotech), and co-transfected into protoplasts isolated from rosette leaves collected from ~4-week-old wild type Arabidopsis plants. Transfected protoplasts were incubated under darkness at room temperature for 20–22 h, and then GUS activities were measured by using a Synergy^TM^ HT microplate reader (BioTEK).

### GUS staining

GUS activity was monitored by staining seedlings and different organs of adult *bHLH129p:GUS* transgenic Arabidopsis plants with X-gluc (5-bromo-4-chloro-3-indolyl-β-D-glucuronide, Rose Scientific Ltd.) using the procedure described previously^49^.

### Microscopy

Photographs of Col wild type and transgenic Arabidopsis seedlings were taken under a Motic K dissection microscope equipped with an EOS 1100D camera. GFP florescence of *bHLH129-GFP* transgenic seedlings was examined, and photographs were taken under an Olympus FV1000 confocal microscope.

## Additional Information

**How to cite this article**: Tian, H. *et al.* An ABA down-regulated bHLH transcription repressor gene, *bHLH129* regulates root elongation and ABA response when overexpressed in Arabidopsis. *Sci. Rep.* **5**, 17587; doi: 10.1038/srep17587 (2015).
